# Lung ultrasound at discharge predicts outcomes in heart failure: a pilot study

**DOI:** 10.2459/JCM.0000000000001613

**Published:** 2024-03-22

**Authors:** Andrea Perillo, Christian Basile, Ilaria Fucile, Francesco Rozza, Nicola De Luca, Costantino Mancusi

**Affiliations:** Department of Advanced Biomedical Science, Federico II University Hospital, Naples, Italy

## Letter to the Editor

Lung ultrasound (LUS) is a valid tool for the assessment of pulmonary congestion in patients with heart failure. However, whether LUS congestion predicts clinical outcomes in heart failure has yet to be widely validated. We investigated the presence of an association between the grade of residual pulmonary congestion assessed by LUS at hospital discharge and 3-month clinical outcomes in patients with heart failure.

## Materials and methods

A prospective observational study was conducted on 85 patients who presented with heart failure during hospitalization in the Cardiac Rehabilitation Unit at the Federico II University Hospital of Naples from March 2021 until September 2022. The study was conducted in accordance with the principles of the Declaration of Helsinki. All participants signed the informed consent before participating.

LUS was performed at hospital discharge using LUS score as an ultrasound indicator of pulmonary congestion. To calculate the LUS score, both hemithoraxes were topographically divided into six regions, each of which was assigned a score from 0 to 3: 0, 0–3 B-lines; 1, >3 B-lines; 2, confluent B-lines; 3, white lung.^1^

Clinical, echocardiographic, and laboratory values were collected at admission and hospital discharge. CKD was defined as an eGFR below 60 ml/min/1.73 m^2^ using the CKD-EPI formula.

Remote monitoring was performed through dedicated phone calls at 90 days after hospital discharge to evaluate new-onset/worsening dyspnea, cardiovascular rehospitalization, angina, and death from cardiovascular causes. We investigated the relationship between available variables and clinical outcomes using a multivariable logistic regression model with converging backward and forward stepwise selection. Multicollinearity was evaluated using the variance inflation factor. All statistical analyses were performed using R (R Foundation). Two-sided *P*-value less than 0.05 was considered significant.

## Results

Baseline characteristics of the enrolled population are summarized in Table 1. The study population was composed of patients with heart failure, 70.5% recovering from an acute myocardial infarction, 20.0% recovering from an acute heart failure event and 9.5% recovering after a complex elective coronary revascularization.

**Table 1:** Baseline characteristics of the enrolled population at hospital discharge

Variables	Overall (*N* = 85)
Age, year [median (IQR)]	71.00 (63.00, 76.00)
Female sex (%)	29 (34.1)
Hypertension (%)	78 (91.8)
Atrial fibrillation (%)	11 (12.9)
Type II diabetes mellitus (%)	30 (35.3)
Chronic kidney disease (%)	13 (15.3)
Chronic obstructive pulmonary disease (%)	17 (20.0)
Heart rate, bpm [mean (SD)]	69.72 (11.03)
SBP, mmHg [mean (SD)]	121.22 (16.73)
DBP, mmHg [mean (SD)]	69.92 (8.04)
SPO_2,_ % [mean (SD)]	96.11 (1.97)
Hemoglobin, g/dl [mean (SD)]	11.86 (1.98)
Sodium, mEQ/l [mean (SD)]	138.51 (2.88)
eGFR, ml/min/1.73 m^2^ [mean (SD)]	67.94 (22.71)
NT-proBNP, pg/ml [median (IQR)]	1277.00 (482.00, 2692.00)
LUS score [median (IQR)]	2.00 (0.00, 4.00)
Ejection fraction, % [mean (SD)]	45.52 (10.56)
Left atrial volume indexed, ml/m^2^ [mean (SD)]	38.02 (14.70)
TAPSE [mean (SD)]	19.95 (4.96)
Pulmonary artery pressure [mean (SD)]	34.26 (10.20)

Median age of the study population was 71 years (63,76), 34.1% were women, with hypertension (91.8%) and Type II diabetes mellitus (35.3%) being the most prevalent comorbidities. Median NT-proBNP was 1277.00 pg/ml [482.00, 2692.00], with a mean ejection fraction of 45.52% ± 10.6% and a median LUS score of 2 (0.00, 4.00).

LUS score at discharge was significantly different between male [1.00 (0.00, 3.25)] and female [3.00 (1.00, 5.00)] (*P* = 0.032) patients with [4.00 (2.00, 7.00)] and without [1.00 (0.00, 4.00)] CKD (*P* = 0.004), and was not significantly different in respect of other baseline variables.

At 3 months, 49 patients reported new-onset or worsening dyspnea; of those, 13 had concomitant angina, 10 had a rehospitalization for cardiovascular causes, and 2 died. Only five patients reported angina without dyspnea.

Univariable linear regressions with baseline characteristics of the enrolled population showed a significant association between the LUS score and NT-proBNP (beta = 0.10 per 500 pg/ml increase, *P* = 0.022), age (beta = 0.82 per 10 years increase, *P* = 0.049), left atrial volume indexed by BSA (beta = 0.65 per 10 ml/m^2^ increase, *P* = 0.027), eGFR (beta = −0.58 per 10 ml/min/1.73 m^2^ increase, *P* = 0.002), and hemoglobin (beta = −0.61 per 1 g/dl increase, *P* = 0.004).

**Fig. 1:**
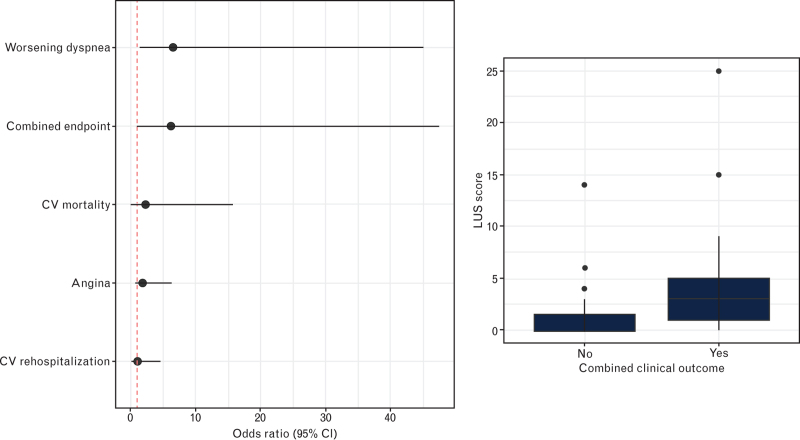
On the left is the logistic regression model of the lung ultrasound score for evaluated 3-month outcomes, and on the right is the boxplot for the value of the LUS score in patients reporting the combined clinical outcome.

Regarding clinical outcome, higher LUS scores at discharge were significantly associated with the composite of new-onset/worsening of dyspnea, cardiovascular rehospitalization, angina, and death from cardiovascular causes [odds ratio (OR) 5.3, 95% confidence interval (95% CI) 1.1–39.6], new-onset or worsening of dyspnea at 3 months (OR 5.5, 95% CI 1.4–36.7, *P* = 0.037), but not with the other clinical outcomes (Fig. 1).

When the combined clinical outcome was evaluated in a multivariable regression model including the LUS score, left ventricle ejection fraction, PAPs, age, sex, and eGFR, the LUS score remained significantly associated with the combined clinical outcome (OR 11.3, 95% CI 2.0–97.8, *P* = 0.01), together with ejection fraction (OR 0.5, 95% CI 0.3–0.87, *P* < 0.01 for every increase of 10% in the ejection fraction) and PAPs (OR 1.9, 95% CI 1.8–9.8, *P* = 0.02).

## Comments

Clinical congestion at discharge has been previously proven to be a valuable predictor of outcomes in patients with heart failure;^2^ however, the best choice to evaluate clinical congestion is still debated.^3^

A recent HFA position paper on the predischarge and early postdischarge management of patients hospitalized for acute heart failure^4^ suggested that during the predischarge phase, a multiparametric evaluation is mandatory in order to minimize persistent congestion and optimize guideline-directed medical therapy. LUS is suggested as a 28-site scanning approach with fewer than 5 B-lines as optimal and fewer than 15 B-lines as acceptable for the congestion evaluation. A recent study from Rosano *et al.*^5^ reported a vulnerable period immediately after an AHF, with the very early events expected in patients discharged before complete relief of congestion, with this being often associated with the pressure to discharge patients early. In this setting, the cardiac rehabilitation unit further strengthens the importance of not discharging the patient until the best possible decongestion has been achieved. LUS is an easy, feasible, and fast evaluation method that adds significant value to the base characteristics of patients with heart failure.^6,7^ The correlation of LUS with eGFR and hemoglobin indicates the ability to select more fragile heart failure patients, like the cardiorenal syndrome phenotype^8^ and iron-deficiency phenotype,^9^ at higher risk of cardiovascular events. This is further proved by its correlation with 3-month new-onset or worsening dyspnea. Albeit no correlation was found with other cardiovascular outcomes, it should be noted that this study was not powered for these outcomes, so these results are only hypothesis-generating. As previously known in literature,^10^^,^^11^ clinical congestion correlates with mortality, with a high morbidity cost on heart failure hospitalization and quality of life; better management with fast and easy-to-use methodologies such as LUS has the potential to reduce the number of heart failure patients discharged with residual congestion.

### Conflicts of interest

There are no conflicts of interest.
